# Pneumopericardium after Laparoscopic Hernia Repair Presents with ST-segment Changes: A Case Report

**DOI:** 10.5811/cpcem.2021.2.51069

**Published:** 2021-03-24

**Authors:** Demi Galindo, Emily Martin, Douglas Franzen

**Affiliations:** * University of Washington School of Medicine, Seattle, Washington; † University of Washington, Department of Emergency Medicine, Seattle, Washington

**Keywords:** case report, pneumopericardium, Hamman’s sign

## Abstract

**Introduction:**

Although rare, iatrogenic cases of pneumopericardium have been documented following laparoscopic surgery and mechanical ventilation. Electrocardiogram (ECG) changes, including ST-segment depressions and T-wave inversions, have been documented in cases of pneumopericardium, and can mimic more concerning causes of chest pain including myocardial ischemia or pulmonary embolism.

**Case Report:**

This unique case describes a patient who presented with chest pain and ST-segment changes on ECG hours after a laparoscopic inguinal hernia repair and who was found to have pneumopericardium.

**Conclusion:**

While iatrogenic pneumopericardium is often self-limiting and rarely requires intervention, it is critical to differentiate pneumopericardium from other etiologies of chest pain, including myocardial ischemia and pulmonary embolism, to prevent unnecessary intervention.

## INTRODUCTION

Pneumopericardium is a rare occurrence and typically results from blunt injury, barotrauma, infection, or abnormal communication between mediastinal structures.^1^ Rare cases have been observed with mechanical ventilation in difficult to ventilate patients, after using high peak airway and positive end expiratory pressures.^2^ While rare, pneumopericardium has been documented after laparoscopy and is thought to occur as gas dissects from the peritoneum into the mediastinum and pericardium via the diaphragmatic hiatus.^3^,^4^,^5^ Electrocardio-gram (ECG) changes including ST-segment depressions have been described in cases of pneumopericardium.^6^ This case describes a patient who presented with pneumopericardium and ST-segment changes on ECG several hours after a laparoscopic inguinal hernia repair, which to our knowledge has only been described once before in the literature.^4^

## CASE REPORT

A 26-year-old woman presented to the emergency department (ED) with chest pain radiating to the left shoulder, following a laparoscopic inguinal hernia repair performed several hours prior to presentation. The patient reported the pain started shortly following the procedure and worsened despite taking acetaminophen and oxycodone, prompting her presentation. She endorsed associated shortness of breath and mild nausea. Her review of systems was negative for associated fevers, chills, cough, abdominal pain, vomiting, or leg swelling. The patient’s medical and surgical history was notable only for appendectomy five years prior to presentation. The patient reported an intrauterine contraceptive device, but no other medications. She reported no allergies. She reported no family history of bleeding or clotting disorders. She reported occasional alcohol and marijuana use.

**Image 1:**
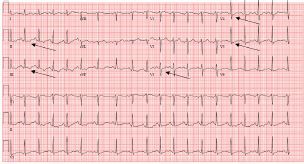
Electrocardiogram demonstrating sinus tachycardia at 115 beats per minute with T-wave inversions in leads II, III (black arrows) and ST-segment depressions in leads V3, V4, V5 (black arrows).

On arrival to the ED the patient was in no acute distress, with initial vital signs notable for heart rate of 114 beats per minute, blood pressure of 110/66 millimeters of mercury, respiratory rate of 20 breaths per minute, oxygen saturation of 100%, and temperature of 36.5 degrees Celsius. An ECG performed in triage demonstrated sinus tachycardia with T-wave inversions in the inferior leads (II, III) and ST-segment depressions in the anterior leads (V3–V5) (Image 1).

On exam, the patient was alert and oriented with moist mucous membranes. Her cardiovascular exam was notable for a regular tachycardia and a holosystolic crunching murmur heard best at the precordium. Palpation of the anterior chest wall revealed crepitus and reproduced her chest pain. Her lungs were clear to auscultation. She had no jugular venous distension or peripheral edema. She had no calf swelling or tenderness, and no discomfort with passive dorsiflexion of either foot. Her abdominal surgical incisions were clean and dry. The remainder of her physical exam was normal.

**Image 2:**
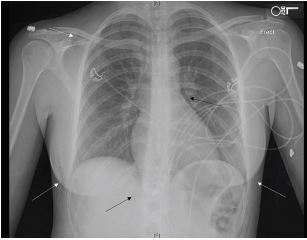
Chest radiograph demonstrating pneumoperitoneum (lower black arrow), subcutaneous emphysema (white arrows), and pneumopericardium (upper black arrow).

Initial workup in the ED included chest radiograph (CXR) and labs including complete blood count (CBC), basic metabolic panel (BMP), troponin, and D-dimer. The patient was given 324 milligrams of aspirin. Her laboratory results were notable for a white blood count of 12.97 thousand cells per cubic millimeter (10^3^ cells/mm^3^) (reference range 4.5–11.0 × 10^3^ cells/mm^3^) with otherwise normal CBC and normal BMP. Her troponin was negative at less than 0.03 nanograms per milliliter (ng/mL) (0–0.3 ng/mL), and her D-dimer was negative at 0.38 micrograms per milliliter (mcg/mL) (0–0.5 mcg/mL). Her CXR demonstrated pneumoperitoneum, subcutaneous emphysema, and pneumopericardium (Image 2).

**CPC-EM Capsule**

What do we already know about this clinical entity?

*Iatrogenic pneumopericardium has been reported after abdominal laparoscopic surgery, presumed secondary to passage of insufflated gas into the mediastinum via the diaphragmatic hiatus*.

What makes this presentation of disease reportable?

*This case describes a patient who presented with pneumopericardium and ST-segment changes hours after laparoscopic hernia repair, which has only been described once before*.

What is the major learning point?

*Although typically benign, pneumopericardium can present with chest pain and electocardiogram changes mimicking more concerning entities like acute coronary syndrome or pulmonary embolism*.

How might this improve emergency medicine practice?

*Emergency medicine clinicians should be familiar with the signs and symptoms of pneumopericardium, and should be able to distinguish pneumopericardium from alternative causes of chest pain*.

Given no evidence of pulmonary embolism, acute coronary syndrome, pneumothorax, pneumonia, or other findings to explain the patient’s chest pain, the subcutaneous emphysema and pneumopericardium were presumed to be the etiology of the patient’s symptoms. Both laparoscopic insufflation and mechanical ventilation were considered as possible causes of her pneumopericardium. Review of the anesthesia records confirmed that the patient was ventilated with low positive end-expiratory pressures, making mechanical ventilation an unlikely culprit. The presence of subcutaneous emphysema suggested laparoscopic insufflation as the likely cause of the pneumopericardium. Insufflated gas was presumed to have traveled through the diaphragmatic hiatus into the pericardial space via the mediastinum. Following administration of aspirin, the patient’s pain improved and her tachycardia resolved. She remained hemodynamically stable throughout her stay in the ED. She was discharged home with follow-up with general surgery and strict return precautions. Unfortunately, she did not follow up in clinic, but is presumed to have recovered without complication.

## DISCUSSION

Pneumopericardium can have significant complications, including cardiovascular and respiratory collapse and cardiac tamponade.^1^ However, iatrogenic pneumopericardium is typically self-limited, resolves spontaneously, and does not often necessitate intervention.^2^,^7^ The diagnosis of pneumopericardium is made by chest radiography. Pneumopericardium can be differentiated from pneumomediastinum on radiography by gas sharply demarcating the pericardial sac around the left ventricle and right atrium (Image 2). Additionally, demarcation of gas can be seen tracing the pulmonary artery and ascending aorta, which form the superior margin of the pericardium. Conversely, gas in pneumomediastinum typically traces the aortic arch and superior vena cava.^1^

Nonspecific ECG changes such as T-wave inversions and ST-segment depressions can be seen in pneumopericardium.^6^ In previous cases of pneumopericardium with ECG changes, patients have been treated for myocardial ischemia or taken for cardiac catheterization unnecessarily.^5^,^6^ In our patient, we considered multiple other underlying etiologies, including pulmonary embolism, pneumothorax, or myocardial ischemia before determining that pneumopericardium was the likely etiology of her symptoms. Differentiating between the pneumopericardium and other etiologies of chest pain is critical to prevent unnecessary intervention.

Hamman’s sign was first described by Louis Hamman in 1937 as a crunching, bubbling, clicking, or popping holosystolic murmur present on cardiac auscultation. It is postulated to be due to physical displacement of air-filled tissues with each heartbeat, and has been described as audible without the use of a stethoscope.^8^ Hamman first described the finding in a case of pneumomediastinum, and a review of literature suggests that Hamman’s sign is found in roughly 30% of patients with spontaneous pneumomediastinum.^9^,^10^ Hamman suggested the finding to be pathognomonic for pneumomediastinum, but the murmur has also been described in cases of left-sided pneumothorax.^11^ Although the test characteristics of this clinical finding have not been described, Hamman’s sign is likely highly specific for pneumomediastinum or pneumothorax, and if auscultated should prompt further investigation.

## CONCLUSION

Pneumopericardium is a rare diagnosis, and can present with chest pain and ECG changes mimicking more concerning diagnoses such as pulmonary embolism or myocardial ischemia. Differentiating between pneumopericardium, which is often self-limiting and typically does not require intervention, and other more concerning etiologies of chest pain is therefore critical. A history of recent laparoscopic surgery or mechanical ventilation should alert the clinician to the possibility of iatrogenic pneumopericardium. Hamman’s sign, a crunching holosystolic murmur due to displacement of air-filled tissue, is likely highly specific for pneumomediastinum and should prompt further investigation. Chest radiography is diagnostic, and will demonstrate gas demarcating the pericardial sac.
